# Narratives and Images Used by Public Communication Campaigns Addressing Social Determinants of Health and Health Disparities

**DOI:** 10.3390/ijerph9124254

**Published:** 2012-11-22

**Authors:** Christopher E. Clarke, Jeff Niederdeppe, Helen C. Lundell

**Affiliations:** 1 Department of Communication, George Mason University, 253 Research Hall, 4400 University Drive MS 3D6, Fairfax, VA 22030, USA; 2 Department of Communication, Cornell University, Ithaca, NY 14850, USA; Email: jdn56@cornell.edu; 3 The Hartman Group, Inc., Bellevue, WA 98005, USA; Email: helen.c.lundell@googlemail.com

**Keywords:** social determinants of health, health disparities, communication, narratives, visuals

## Abstract

Researchers have increasingly focused on how social determinants of health (SDH) influence health outcomes and disparities. They have also explored strategies for raising public awareness and mobilizing support for policies to address SDH, with particular attention to narrative and image-based information. These efforts will need to overcome low public awareness and concern about SDH; few organized campaigns; and limited descriptions of existing message content. To begin addressing these challenges, we analyzed characteristics of 58 narratives and 135 visual images disseminated by two national SDH awareness initiatives: The Robert Wood Johnson Foundation’s Commission to Build a Healthier America and the PBS-produced documentary film *Unnatural Causes.* Certain types of SDH, including income/wealth and one’s home and workplace environment, were emphasized more heavily than others. Solutions for addressing SDH often involved combinations of self-driven motivation (such as changes in personal health behaviors) along with externally-driven factors such as government policy related to urban revitilization. Images, especially graphs and charts, drew connections among SDH, health outcomes, and other variables, such as the relationship between mother’s education and infant mortality as well as the link between heart disease and education levels within communities. We discuss implications of these findings for raising awareness of SDH and health disparities in the US through narrative and visual means.

## 1. Introduction

The evidence base on how social determinants of health (SDH) shape population health outcomes, disease prevalence, and health disparities is substantial [1,2,3,4,5]. SDH are political, economic, and social factors that reflect “conditions in which people are born, grow, live, work and age” [6]; examples include housing, workplace, and community resources; social support; education; income; and discrimination [7]. Their impact on health is multi-faceted, ranging from biology to behavior. Stress caused by economic insecurity can depress hormonal, neurological, and immune systems [8]. Social, political and economic conditions shape the built and natural environment, which influence opportunities to engage in healthy behaviors and seek health care [9]. By virtue of living and working within comparatively unhealthy physical, economic, and social environments, socially disadvantaged populations in terms of education, income, race, and/or political influence face challenges in improving health [10] even when access to medical care and motivations to engage in healthy behavior are high [11].

Public health researchers and advocates have focused on raising public awareness and mobilizing support for policies to address SDH [12] in areas such as early childhood education, obesity prevention, improved nutrition in schools, and reducing income inequality [1]. Such research is important because SDH are influenced by how communities, states, and countries distribute financial and social resources through policy decisions [8], and policymakers devote greater attention to issues that are salient to the public [13]. These efforts have included persuasive messages targeting policymakers, private sector leaders, and citizens [14]. Scholars from communication, public health, and other fields have also argued that narratives and visuals are effective tools for communicating about SDH and health disparities and achieving the aformentioned awareness and advocacy goals. In particular, they are well-suited to illustrating how they shape health outcomes and disparities and how policy interventions can address these issues [12]. However, while research on communicating about SDH and health inequities is growing, the topic remains underexplored. In this article, we articulate relevant communication challenges. Also, to begin addressing these challenges, we analyze narrative and visual message characteristics disseminated by two SDH awareness initiatives in the U.S. We seek insight into choices made in these campaigns related to communicating health determinants and disparities and potential implications for future efforts in this area.

### 1.1. Communicating about SDH: Challenges and Opportunities

Narrative is defined as a “structured, coherent retelling of an experience” that “has an identifiable beginning, middle, and end… provides information about scene, characters, and conflicts; raises unexpected questions or unresolved conflict; and provides resolution” [15]. Drawing on theories of narrative persuasion [16,17,18,19], such stories may help effectively communicate about SDH and health disparities in several areas. First, they have the ability to raise awareness of these issues; examples include diseases such as diabetes [20] and obesity [21,22]. Second, they can help inform beliefs about who is responsible for addressing these issues (*i.e.*, society *versus* the individual). Individual responsibility for engaging in healthy behaviors and seeking medical care dominate media coverage of health [23,24] and public perception. Although Americans are increasingly aware of the SDH-health connection [25], it is still seen as comparatively less important (and less worthy of efforts to change policy) than improving personal health practices [26]. These perceptions are a function of beliefs about who is responsible for causing and solving poor health outcomes and disparities [27,28]. These attribution judgments matter, as belief in social, environmental, and economic factors that shape health is associated with stronger support for policy targeting SDH. Third, narratives can help mobilize support for policy initiatives by influencing these judgments [12]. 

Fourth, they may also be useful for illustrating broader health trends using individual exemplars [29]—for example, how one person’s experience with SDH is emblematic of how SDH shape population health overall. Finally, they can help communicate complex interactions among personal behavior, SDH, health outcomes, and policy interventions [30]. While Americans have a narrow conception of policies that address SDH and health disparities [12], support may depend on appreciating that health is a complex system shaped by behavior, health care coverage, and dynamic social, economic, and environmental factors and policies [30]. These phenomena can serve as “characters” in a story about what shapes health, how/why, and what can be done to achieve optimal health outcomes through attention to these factors. However, despite these advantages, the power of storytelling also involves potential drawbacks. Individual exemplars can be afforded a level of attention or concern that is disproportionate to available statistical evidence (e.g., leading one to believe that a particular group suffers a greater disease burden than is actually the case) [31,32,33]. They can also distract the reader from the intended persuasive message if one focuses instead on incidental story details [15]. 

Research on visual images has focused on their ability to document that an event occurred [34]; convey health risk information [35] (such as including variables like SDH, health behavior, and health outcomes); provide concrete illustrations of story events; and facilitate attitude and behavior change. For example, a picture of a protagonist amid a backdrop of an impoverished neighborhood could visually highlight how different elements of one’s surroundings (*i.e.*, crime, presence of sidewalks, and access to food stores) shape one’s health. These qualities have led researchers to compare the persuasive effectiveness of visual *versus* textual information [30,36]. Juxtaposing two or more images, moreover, can serve a variety of persuasive functions, including (1) inviting causal interpretations, (2) suggesting generalizations, (3) highlighting contrasts or differences, and (4) emphasizing analogies or similarities [31]. For example, juxtaposed maps could help convey causal relationships between two or more variables [37] (such as diabetes prevalence in a community as a function of the education level of its residents). Such relationships can also be represented by visuals incorporating statistics (*i.e.*, a bar chart depicting infant mortality as a function of a mother’s education level and race).

### 1.2. Existing SDH Awareness Campaigns

We analyzed narrative and visual message characteristics disseminated by two recent SDH awareness initiatives in the US Both developed messages about SDH and health disparities aimed at policymakers and the public, such as “where we live, work, and play can have a greater impact on how long and well we live than medical care” [14]. They also featured narratives and images illustrating the SDH-health connection and relevant interventions. However, to date there has been no systematic description of the content of these messages. We describe each campaign below, starting with the RWJF Commission to Building a Healthier America (“The Commission”): a nonpartisan group of representatives from academic institutions, government, and non-profit organizations pursuing the goal of “(raising) awareness and (achieving) greater consensus…about the social factors that affect health and the need to act across sectors to improve the health of all Americans” [38]. The Commission (1) produced several reports on the SDH-health connection; (2) issued a variety of recommendations for policymakers and private sector leaders related to school lunch programs, physical activity in schools, and becoming a smoke-free nation; and (3) developed key messages, couched in narrative and visual terms, to communicate this information. Twenty-one stories were developed about individuals faced with and responding to SDH. Graphs and charts were included in a majority of reports to help draw the viewer’s attention to broader themes regarding health determinants and disparities.

*Unnatural Causes* was a four-hour Public Broadcasting Service (PBS) documentary produced by California Newsreel in 2008. Seven chapters followed individuals, families, and communities as they responded to health issues tied to social, economic and political circumstances. The film featured narratives and visual images about SDH. The accompanying website also allowed viewers to post their own health-related stories and experiences. 

## 2. Methods

In the summer of 2009, we screen-captured narratives produced by The Commission (for a full list, visit http://www.commissiononhealth.org/Stories.aspx) as well as all reports from which we identified graphs, charts, and other images (see http://www.commissiononhealth.org/Publications.aspx). For *Unnatural Causes,* we identified stories by reviewing transcripts of the film and screen-capturing ones that the public had posted to the website (see http://www.pbs.org/unnaturalcauses/share_your_story.htm). Finally, we identified graphs, charts and map images by watching the DVD. Each of the 58 stories and 135 images across both campaigns were the unit of analysis. 

We developed a quantitative coding scheme (available upon request) to analyze the content of narratives and images. Narrative coding variables, indicators, and examples are provided in Table 1. Our coding involved identifying a “plot” involving some kind of action (*i.e.*, a health issue linked to SDH) and attempted “resolution” (*i.e.*, addressing SDH through policy change). Specifically, we identified health issues protagonists faced as well as types of SDH and impacts on health. For the latter, we consulted publications by The Commission, *Unnatural Causes,* and academic literature. We also reviewed research on analyzing narrative and visual representations of health messages [37,39]. Finally, we grouped policies to address these impacts into categories based on who or what initiated them, and who benefited from them. “Self-driven decisions” involved personal behavior change, such as a protagonist deciding to engage in physical activity. “Self-driven interventions”involved a protagonist engaging in behavior that benefits other people, such as forming a community health support group. “Externally-driven interventions” were implemented by outside actors, such as non-profit organizations sponsoring urban renewal projects. We also coded for the presence of one or multiple examples within the same story. A list of visual coding variables, indicators, and examples is provided in Table 2.

We focused on types of variables the campaigns attempted to convey related to the SDH-health connection. Specifically, we identified the gender and race/ethnicity of each character(s) in photos that accompanied narratives from The Commission. For maps, graphs, charts, and other visuals, our variables included demographic characteristics like gender; health outcomes; health behaviors; and SDH (using the same operational definition as for the narratives). To examine juxtaposed images, defined as two visuals side-by-side so as to suggest an association, we used four categories described in previous work: inviting causal interpretations, suggesting generalizations, highlighting contrasts or differences, and emphasizing analogies or similarities [34]. For example, two maps could be presented in which one displays the prevalence of a health outcome (such as obesity) in a given area and the other displays the presence of area parks; these visuals together would arguably represent an attempt to causally link obesity to environmental factors such as access to places to exercise. 

We developed a draft codebook and met with two coders to review the material and address questions. We then trained them using approximately 40% of the total narrative and visual sample. We spent approximately 20 h on training. The coders proceeded to independently analyze the remaining 60% of the sample. At the conclusion, we calculated percent agreement. Narratives averaged 79%, and visuals averaged 83% agreement, consistent with previous studies assessing narrative and images in other contexts [39]. Discrepancies were addressed by having coders discuss differences and come to consensus, and we report consensus codes in this article. We did so because the small number of narratives (n = 58) and photos, graphs, charts, and other images (n = 135) meant that we had few available for both coder training and reliability assessment. Also, when assessing inter-rater reliability using statistics such as kappa, the small sample size and conservative nature of the test resulted in situations where high percent agreement nonetheless resulted in low reliability. Nonetheless, we believe that rigourus development of the coding scheme and consensus coding ensured reliable and valid data.

## 3. Results

### 3.1. Narratives

We coded 58 naratives: 21 from The Commission and 37 from *Unnatural Causes.* In Table 1, we present the frequency of each indicator across all narratives and within each campaign. Health issues were mentioned in 56 of 58 narratives. General references to health were the most frequently cited, followed by non-disease conditions such as stress, and physical diseases such as cardiovascular disease and diabetes.

**Table 1:** Narrative Coding Variables, Indicators, Examples, and Frequencies.

Variable	Indicator	Description and Examples	Frequency—The Commission (n = 21; % within)	Frequency—Unnatural Causes (n = 37; % within)	Total (n = 58; % overall)
*Number of Protagonists*	Single protagonist	Only one protagonist discussed (“Meet James Moon”)	16 (77%)	28 (76%)	44 (76%)
	Multiple protagonists	≥ 2 protagonists discussed (“Meet the Elkins family”)	5 (23%)	7 (19%)	12 (21%)
	Other	Protagonist unclear	0	2 (5%)	2 (3%)
*Heath issue(s)*	Physical diseases	Diabetes; cardiovascular diseases	9 (43%)	22 (60%)	31 (53%) ^a^
		*When it came to warding off the threat of diabetes that has plagued her family, Kathleen Dolezal got a boost…*			
*56 of 58 narratives mentioned at least one health issue*	Non-disease conditions	Explicit references to stress	8 (38%)	24 (65%)	32 (55%) ^a^
		*Silva’s workplace…only allowed two 15-minute breaks and a half-hour lunch. Silva needed to express milk at least three times each day to… feed her baby…..“I had to be back in 15 minutes,” said Silva. “It was very stressful.”*			
*Heath issue(s)* *(continued)*	Emotional/mental conditions	Depression; feeling depressed	5 (24%)	6 (16%)	11 (19%) ^a^
		*Two times a week, Chuck rides one of the bike paths…it kills whatever **depression** you have.*			
	Emotional well-being	Worry and anxiety (not clinically diagnosed)	11 (52%)	14 (38%)	25 (43%) ^a^
		*Malcolm is the sort of teen who makes everything look easy… [but]Malcolm had difficulty opening up with adults…he worried about his appearance…*			
	Lifestyle issues	Weight gain/loss/struggle; obesity; sense of fulfillment (or not)	11 (53%)	8 (21%)	19 (33%) ^a^
		*Retirement didn’t quite suit Paris… even though she continued to lead an active life and maintained a “glass half-full” attitude… she felt something was missing. “There was an emptiness. I wasn’t ever quite satisfied.”*			
	General health	General mentions of “health”,” illness,” *etc*.	14 (66%)	20 (54%)	34 (59%) ^a^
		*Ed Fendley touts walking and biking as good for the bottom line. …But as a parent, Ed sees even greater value….“One of the motivations is health,” he says.*			
*Social determinants of health (SDH)*	Workplace	Workplace dangers, resources; unemployment	5 (24%)	15 (41%)	20 (35%) ^a^
		*Silva’s workplace…only allowed two 15-minute breaks and a half-hour lunch. Silva needed to express milk least three times each day to…feed her baby…..“I had to be back in 15 min,” said Silva. “It was very stressful.”*			
*54 of 58 narratives mentioned at least one SDH*	Income/ poverty/ wealth	Income; ability to afford things; economic status	8 (38%)	20 (54%)	28 (48%) ^a^
		*I grew up in the projects in NW DC….We lived miles away from a grocery store and did not own a car. We walked (to save bus fare) once or twice a month and dragged mostly vegetables (fruit was too high) home in a cart….*			
*SDH (continued)*	Education	References to formal education	3 (14%)	8 (22%)	11 (19%) ^a^
		*The effects of social factors, such as income, education, and neighborhood resources are particular dramatic [on health]*			
	Knowledge/Skills	References to a lack of knowledge or skills related to health	4 (19%)	0	4 (7%) ^a^
		*But it hasn’t been easy [for Norman to lose weight]. “First of all, I don’t know how to cook all of these things,” she says, referring to her doctor’s dietary advice.*			
	Home/housing	Poor/unsafe housing; homelessness	10 (47%)	9 (24%)	19 (33%) ^a^
		*In daughter Ananaya’s short life, she’d experienced a litany of maladies.. …But last winter, after moving into the newly-renovated Viking Terrace Apartments, Ananaya did not get sick once ….[They] are living proof that one’s environment has a direct impact on health….their three-bedroom unit in southwest Minnesota includes air conditioning, exhaust fans in the kitchen and bathrooms and no mold anywhere*			
*SDH (continued)*	Discrimination	Based on age, race, gender, etc	1 (5%)	7 (19%)	8 (14%) ^a^
		*I am a gay male who has been under tremendous stress throughout my life and subject to…discrimination…..*			
	Neighborhood context	Opportunities for physical activity; crime; pollution	10 (48%)	16 (44%)	26 (45%) ^a^
		*When it came to exercise, getting outdoors was a risky proposition in **crime-ridden neighborhoods with few safe parks and playgrounds**.*			
	Social support	Social support; sense of community	5 (24%)	6 (16%)	11 (19%) ^a^
		*Wolff, an energetic and dedicated night-shift nurse, [decided] to kick her “addiction” to food and start living healthy…Support for Wolff’s journey towards better health came from…an unexpected outpouring of support from her co-workers.*			
*Processes of Change:* *Personal Decisions and/or Interventions*	Self-driven decisions	Exercise (*i.e.*, riding bike and running); initiated by protagonist, benefits accrue to that individual	2 (10%)	2 (5%)	4 (6%)
		*Staying healthy by eating right and getting exercise has a lot to do with individual choices. But communities can help—or hinder, says James Moon, an Arlington County, VA., resident….when he got the chance, he moved to East Falls Church…the county has 86 miles of jogging-biking trails and 200 public parks and playgrounds.*			
*28 of 58 narratives mentioned at least one process of change*	Self-driven interventions	Protagonist forms a local community non-profit; forms a support group; benefits accrue to others beyond protagonist	0	1 (3%)	1 (2%)
		*In 2005, [Chuck also] **formed Broadway Public Arts**, which sponsors street fairs, concerts, parades and holiday house decorating contests. The events serve several purposes, including fostering better race relations in the multi-cultural neighborhood.*			
*Processes of Change (continued)*	Externally-driven interventions	Employer initiates a health program; non-profit organizations work on urban renewal projects; employers provide childcare services	3 (14%)	3 (8%)	6 (10%)
		*Organizations such as the nonprofit **Slavic Village community development corporation** and the **Robert Wood Johnson Foundation’s Active Living by Design program** have helped **lead an urban renewal** that places a big emphasis on health and well-being.*			
	Self-driven decisions and externally-driven interventions together	Joining a workplace health initiative (employer sponsors, employee decides to partake)	11 (52%)	1 (3%)	12 (20%)
		*Dolezal signed up for a 12-week program called N-Lighten that the state made available to its employees. She lost 17 pounds and substantially reduced her blood-glucose levels, which had been cause for concern…*			
	All three types	Elements of all three types (*i.e.*, joining a workplace health initiative and forming a workplace support group independent of that initiative)	5 (24%)	0	5 (9%)
	None present	N/A	0	30 (82%)	30 (52%)

^a^ Column totals for indicators do not equal 100% because indicators were not mutually exclusive; multiple ones could be—and, at times, were—present in the same story.

**Table 2:** Visual Coding Variables, Indicators, Examples, and Frequencies.

Variable	Indicator	Description and Examples	Frequency		
			The Commission	Unnatural Causes	Total
*Photos (The Commission only)*	Only females present	At least one female character present only	21 (47%)	N/A	21 (47%)
	Only males present	At least one male character present only	16 (36%)	N/A	16 (36%)
	Females and males present	At least one male and female character present	8 (17%)	N/A	8 (17%)
	Individual featured is White	N/A	18 (40%)	N/A	18 (40%)
*(n = 45)*					
	Individual featured is Black	N/A	20 (44%)	N/A	20 (44%)
	Individual featured is Hispanic	N/A	5 (11%)	N/A	5 (11%)
	Black and White featured	N/A	2 (5%)	N/A	2 (5%)
*Variables Depicted in Maps, Graphs, and Charts*	Health outcomes	Diseases, death rates, life expectancy, *etc*.	5 (8%) ^a^	8 (29%) ^b^	13 (14%) ^c^
	SDH	Income and education	5 (8%)	2 (7%)	7 (7%)
	Health behaviors	Smoking, dieting, *etc*.	1 (1%)	0	1 (1%)
	Demographic variables	Age, sex, race, ethnicity	0	2 (7%)	2 (2%)
	Health outcomes & SDH	Infant mortality as a function of mother’s education	21 (33%)	9 (35%)	30 (33%)
	Health outcomes & demographics	Age-adjusted mortality of different racial groups	4 (6%)	2 (8%)	6 (6%)
	SDH & demographics	Accumulated wealth of different racial groups	11 (17%)	0	11 (12%)
*(n = 90)*					
	SDH and health behaviors	Performance of health behaviors as a function of education	1 (2%)	0	1 (1%)
	Health outcomes, SDH, and health behaviors	Health as a function of poverty and performance of health behaviors	3 (5%)	0	3 (3%)
	Health outcomes	Diseases, death rates, life expectancy, *etc*.	5 (8%) ^a^	8 (29%) ^b^	13 (14%) ^c^
	SDH	Income and education	5 (8%)	2 (7%)	7 (7%)
	Health behaviors	Smoking, dieting, *etc*.	1 (1%)	0	1 (1%)
	Demographic variables	Age, sex, race, ethnicity	0	2 (7%)	2 (2%)
*Presence of Juxtaposition in Maps, Graphs, and Charts*	Health outcomes & SDH	Infant mortality as a function of mother’s education	21 (33%)	9 (35%)	30 (33%)
	Health outcomes & demographics	Age-adjusted mortality of different racial groups	4 (6%)	2 (8%)	6 (6%)
	SDH & demographics	Accumulated wealth of different racial groups	11 (17%)	0	11 (12%)
	SDH and health behaviors	Performance of health behaviors as a function of education	1 (2%)	0	1 (1%)
	Health outcomes, SDH, and health behaviors	Health as a function of poverty and performance of health behaviors	3 (5%)	0	3 (3%)
	Health outcomes, SDH, and demographics	Infant mortality as a function of mother’s education and racial group	11 (17%)	4 (15%)	15 (17%)
	No variable present	N/A	0	1 (4%)	1 (1%)
*n = 3*					
	Present	N/A	1 (2%) ^d^	2 (7%) ^d^	3 (3%) ^d^
	Inviting causal interpretations	Map of heart disease rates in the US presented next to a map of education levels (*i.e.*, percentage of population in each state with a college degree)	3 (100%)	0	3 (100%)
	Suggesting generalizations	N/A	0	0	0
	Highlighting contrasts or differences	N/A	0	0	0
	Emphasizing analogies or similarities	N/A	0	0	0

^a^ Population size for the Commission was 64 for the remainder of this column. ^b^ Population side for *Unnatural Causes* was 26 for the remainder of this column. ^c^ Total population size was 90 for the remainder of this column. ^d^ Total population size was 3 for the remainder of this column.

Furthermore, the vast majority of narratives (54/58) mentioned at least one SDH, including income/poverty/wealth (n = 28), such as references to income and economic status; neighborhood context (n = 26), including opportunities for physical activity in one’s neighborhood as well as crime and environmental pollution; unsafe workplace conditions (n = 20); and home/housing factors (n = 19), such as poor, unsafe, and unhealthy housing. Finally, nearly half of narratives (n = 28) described strategies to address these impacts, of which most (n = 21) came from The Commission. The most common type was self-driven decisions in tandem with externally-driven interventions, which appeared in 12 narratives. 

To illustrate these findings in more detail, we highlight a specific narrative from The Commission website that chronicled the health problems faced by Abang Ojullu, a recent immigrant to the US, and her daughters (visit http://www.commissiononhealth.org/MiniStory.aspx?story=60086 for the full story). The story began with an overview of the health problems faced by Abang’s daughter:

*Abang Ojullu remembers all too vividly the day she put her eldest daughter on a small ambulance jet bound for Sioux Falls. The child’s asthma attack was too severe for doctors in rural Worthington, MN to treat. In daughter Ananaya’s short life, she’d experienced a long litany of maladies. Some winters, the girl’s asthma was compounded by pneumonia.*

The role of unhealthy housing in which the family previously lived was then introduced:

*Research has shown that low-income families are particularly vulnerable to environmental hazards in their homes, such as lead paint, mold, crowding and rodents and other pests, as well as problems such as water leaks, poor ventilation and dirty carpets that can lead to an increase in mold, mites and other allergens associated with poor health. More than 20 million Americans have asthma, and about 40 percent of diagnosed asthma among children is believed to be a result of residential conditions.*

Finally, the story described how, after the family moved to a “healthier” home, the child’s health problems improved:

*But last winter, six months after moving into the newly-renovated Viking Terrace Apartments, Ananaya did not get sick once, her mother says…Unlike previous residences, their three-bedroom unit in southwest Minnesota includes air conditioning, exhaust fans in the kitchen and bathrooms and no mold anywhere. The apartment complex also added a playground with benches for the adults…The physical improvements, along with a health education campaign, are part of a joint project aimed at demonstrating the health benefits of green building principles. Participants include the National Center for Healthy Housing, the Blue Cross and Blue Shield of Minnesota Foundation, Minnesota Green Communities, the Southwest Minnesota Housing Partnership and local and state governments.*

We coded this passage as a self-driven decision in tandem with an externally-driven intervention. The decision to move was arguably voluntary in that the family had a choice about whether to do so. This decision, however, was facilitated by the availability of the housing complex described above, which was made possible by entities beyond the protagonists. 

### 3.2. Visuals

Table 2 provides a full overview of visual coding results. We coded 135 images, including 45 photos associated with The Commission narratives, eight line graphs, 49 bar charts, 14 other types of graphs/charts, eight lists, four tables, and seven maps. The majority of photos featured only one individual. In terms of demographics, females were present in more photos than males. Most stories featured either a White/Caucasion or a Black/African-American character. The entire complement of photos accompanying the narratives can be found by visiting http://www.commissiononhealth.org/Stories.aspx. 

Three types of variables emerged most often in the non-photo sample (n = 90). The first was health outcomes and SDH together. For example, a bar chart in a Commission report (for a reprint, please visit http://www.commissiononhealth.org/PDF/momedinfmt.pdf) displayed infant mortality as a function of mother’s education, with the caption “babies born to mothers who have not finished high school are nearly twice as likely to die before their first birthdays as babies born to college graduates.” The second type was a tripart combination of health outcomes, SDH, and demographics. Another report, for example, included a bar chart illustrating perceptions of poor/fair health as a function of family income level and racial/ethnic group (for a reprint, visit http://www.commissiononhealth.org/PDF/inchlthxeg.pdf). The caption stated that “differences in health status by income do not simply reflect differences by race or ethnicity; differences in health can be seen within each racial or ethnic group. Both income and racial or ethnic group matter.” The chart suggested that whether Black, Non-Hispanic; Hispanic; or White, Non-Hispanic, lower income households tend to report poorer health and vice-versa.

Finally, juxtaposition was present in three of the seven maps, but in none of the other visuals. We coded all three as inviting causual interpretations. For example, one map presented data on education level in each county in the US (measured by the percentage of the population with a college degree) along with the caption that “educational attainment among adults varies markedly across different regions of the country.” On the next page, a second map presented data on rates of heart disease in these same counties; the accompanying caption stated that “disease varies geographically. For example, higher rates of death due to heart disease are often seen in areas where fewer adults have college educations” (to access both maps, visit http://www.commissiononhealth.org/PDF/Obstacles ToHealth-Highlights.pdf, pp. 20 and 21). 

## 4. Discussion

In this article, we articulated communication challenges associated with raising awareness of and mobilizing action regarding SDH and health disparities. To address these challenges, we presented data on narrative and visual message characteristics disseminated by two national SDH awareness initiatives in the U.S. In this section, we explore implications of these findings.We begin by discussing the communication implications of our narrative findings, which we couch in the context of limitations of our analysis. We did not test the persuasive impact of these narratives or theory related to narrative persuasion. However, this analysis serves an important purpose. As part of efforts to study the persuasive impact of narratives about SDH and health disparities [30,40] in contexts such as HIV/AIDS and other infectious diseases [41]; chronic diseases such as diabetes [20]; and obesity [21], our analysis is positioned to describe features of these messages and help determine what facets could potentially lead to desired outcomes. In particular, it draws on relevant theory to argue that choices these and future campaigns make in communicating about SDH and health disparities using stories, such as the type of health determinants, health outcomes, and policy interventions highlighted, has important implications for convincing citizens and other stakehlolders that (1) SDH contribute significantly to adverse health outcomes and disparities; (2) responsibility for addressing SDH rests both in individual behavior as well as social (*i.e.*, governmental) policy; and (3) there are policy solutions to tackle these issues that warrant and need support. Relevant theoretical perspectives include framing of issue responsibility, individual exemplars, and other domains. 

Also, we focused on The Commission and *Unnatural Causes,* which were, to our knowledge, the only two large-scale, US-based campaigns that were strategically designed to communicate the importance of SDH as of 2009 (when we conducted the analysis). Although other, non-US based initiatives such as the World Health Organization’s 2008 report titled *Closing the Gap in a Generation—Heath Equity through Action on the Social Determinants of Health* [6] have also been published and gained well-deserved notoriety, they did not feature narrative evidence. To the extent initiatives similar to The Commission or *Unnatural Causes* are produced in the future (such as the 2012 HBO documentary *Weight of the Nation* (http://theweightofthenation.hbo.com/), which explored behavioral and social-structural factors driving obesity in the US), research could compare them with our sample. 

Our analysis suggests several opportunities and challenges for using narratives to communicate about SDH and health disparities. Communication scholars have written extensively about the “right” formula for a “good” (*i.e.*, persuasive) narrative, citing factors such as a coherent plot with a clear beginning, middle, and end as well as engaging characters [17]. We view the health issues faced by protagonists in the narratives as a key element of story plot. Both initiatives emphasized generic and specific health issues, with the former (using examples indicative of generally “poor health”) comparatively more numerous. In terms of the latter, cardiovascular-related conditions such as heart attack and stroke were prominently emphasized; these conditions are among the the leading cause of death in the US [42] and worldwide [43]. To our knowledge, existing research on the design and testing of persuasive SDH messages have not given as much attention to types of health issues to which SDH relate. Future research should examine whether it is desirable to highlight connections among SDH, health disparities, and health outcomes through attention to specific or general conditions (or both). Such research may draw on the concept of identification: the extent one is able to imagine being, behaving like, and empathizing with a character in a story; it is an important determinant of narrative persuasion [18,44]. We suggest that the ability of narratives to raise awareness of and mobilize policy support around SDH and health disparities may depend, in part, on the extent people can “identify” with the health conditions that are argued to shape/be shaped by these factors. Both generic and specific health issues may have advantages and disadvantages. For example, narratives that employ specific isssues could be effective in conveying awareness because they touch on potentially well-known conditions and causes of death. A generic focus on “health” could allow campaigns to stress that conditions where people live, work, and play shape overall well-being. 

Another important story element is SDH themselves: arguably the “villains” preventing protagonists from achieving good health. Both campaigns focused on SDH that have received considerable attention in health policy discourse and are particularly salient in the public vernacular: income/poverty/wealth, neighborhood context, and workplace/housing-related issues. For example, a 2007 national survey [25] found that 78% of Americans agreed that “it is important to make sure health differences between groups of people in this country no longer exist because of factors such as income,” and 82% felt that “living in a safe neighborhood can have a positive influence on a person’s health.” However, there was comparatively little focus on other SDH like racial discrimination, education, and social support, and survey research suggests that these examples are less salient than their aforementioned counterparts. Robert *et al.* [45] found that less than half of Wisconsin adults believed that neighborhood safety (41.9%), quality of housing (33.2%) and education (33.7%) have strong effects on a person’s health. Education ranked lower in awareness than one’s environmental surroundings. Overall, SDH garnered less awareness than factors like personal health practices (*i.e.*, diet and exercise) (84.6%), health insurance (75.3%), and access to affordable health care (69.8%). 

SDH advocates do not simply seek to show that social, political, economic, and environmental factors are important drivers of health; they also strive to communicate that health is substantially more complex than an individuals’ willpower or health care coverage alone. SDH may not be created equal in terms of the extent they endanger public awareness or concern. It is possible that some SDH are more likely than others to conjure feelings of personal responsibility and deficiencies as opposed to an appreciation of social-structural considerations [46]. For example, education can involve beliefs about the availability of quality schools and/or that an individual simply needs to work harder to learn. The former arguably represents a structural factor while the latter speaks to personal motivation. These beliefs, moreover may also impact public views on who is responsible for addressing these health determinants; people may be less likely to support government policies to address SDH if they believe that those determinants have roots in personal deficiencies, can be overcome through behavior change, or serve to stigmatize certain groups. Research suggests, for example, that emphasizing negative health outcomes or disparities among lower socioeconomic groups is viewed, at least by some individuals, as reifying that “lower status” individuals cannot improve their health [30,47]. 

Finally, nearly half of narratives described strategies to address SDH impacts, of which most (21 out of 28) came from The Commission. A potential explanation may be the contrasting advocacy approaches among the two organizations. The Commission issued a variety of recommendations for addressing SDH, many of which also appeared, in some form, in the narratives. In contrast, *Unnatural Causes* featured a separate advocacy component. A page on the film’s website (http://www.unnaturalcauses.org/what_you_can_do.php) described how organizations could use the film to educate, organize, and advocate for change. The idea was that the film would raise awareness of SDH and their consequences, while later advocacy efforts would focus on changing them. Since our analysis focused on the film, we likely did not capture all potential interventions that factored into this broader advocacy strategy. 

SDH researchers and advocates have stressed the importance of offering solutions, especially within narratives, to issues involving connections among SDH, health outcomes, and health disparities [1,14]. The strategies mentioned in the narratives we examined tended to be a combination of self-driven decisions and externally-driven interventions. These findings reflect a larger debate about the role both of personal responsibility, including health behaviors, and structural factors in shaping health outcomes and disparities and serving as components of persuasive messages. There is a complex, mutualistic relationship between the two. Health behaviors are, in part, consequences of structural factors [48]; for example, lack of convenient food markets in one’s neighborhood can undermine the ability to make healthy food choices. Consequently, SDH-themed messages may need to acknowledge the role of personal choice and healthy (or unhealthy) behavior; the role of “external” structural factors; and how programs can help change these behaviors through attention to those factors. Public health organizations and researchers have begun using a “mixed” approach that acknowledges both personal responsibility/behavior as well as SDH [12,30,49]. Preliminary results point to the ability of such an approach, especially when couched in narrative terms, to increase audiences’ belief that societal actors (such as the government) are responsible for tackling SDH and subsequent health outcomes (such as obesity [21]); increase support for policies targeting SDH and health disparities [49]; and reduce the likelihood that people counter-argue the message that behavior and external factors synergistically affect health [22]. 

In addition, scholars are also examining the role that existing predispositions play in how people react to such information. For example, political ideology is an important moderator of persuasive effects of SDH-themed narratives, with liberal audiences less likely to counter-argue intended messages and conservative audiences more skeptical about how SDH shape health outcomes (as opposed to health behavior alone) [20,21]. There is ample opportunity and necessity to expand the audience pool from citizens to policymakers, given that public support for SDH policy is most effective with policymaker buy-in. Research is beginning to explore policymaker perceptions of health determinants and disparities, especially in the context of specific issues like obesity, as well as views on appropriate and feasible policy solutions [50]. Narrative-based information using a “mixed” approach discussed above may be particularly effective as part of policy briefs [51]. These briefs summarize what is known about SDH and health disparities and what can be done to combat adverse health outcomes. The challenge advocates and researchers face relates not only to the political ideology of individual policymakers but also that they tend to be of a higher socioeconomic and health status compared to the rest of the population (and, thus, may feel no personal stake in health policy) [12]. 

Overall, the synthesis of the individual and the social-structural is challenging: emphasizing SDH without overlooking personal responsibility and promoting responsibility without taking a “blaming the victim” approach. The success of interventions that stress both may hinge, in part, on clearly explaining how a given strategy can produce a certain (presumably health-enhancing) outcome: in other words, not just explaining how/why SDH shape health outcomes and disparities but also how/why an advocated solution “works” to improve health. Recent research suggests that people may be skeptical of a “black box” description of SDH-themed interventions included in narratives such as those used by The Commission and *Unnatural Causes*. For example, they may believe that the situations portrayed do not happen in “normal” life [30,40]; that the solutions are not replicable elsewhere; or that insufficient attention was afforded to how the outcome(s) came to be. Clearly delineating steps through which behavior change, in concert with external policies, achieve positive health outcomes is an important narrative element and warrants future research. 

Our analysis of visuals present in the two campaigns likewise has several limitations. We concentrated on specific sections of website and film that contained still images such as charts, graphs, and maps. We did not code moving images within *Unnatural Causes*, which can involve factors such as camera angle and editing [34]. Moreover, scholars could build on this study by focusing on SDH-themed visuals that have been featured in more recently produced programs, such as HBO’s *The Weight of the Nation*. Nonetheless, our analysis is positioned to describe image features, such as types of variables represented and juxtapositinons used, and help determine what facets could potentially lead to desired outcomes by drawing on relevant theory. Many of the 90 maps, graphs, and charts featured information about a specific health outcome either in isolation or, more frequently, in combination with other variables including SDH and demographics. However, image juxtapositions featuring two visuals side-by-side were relatively rare. In terms of persuasive appeal, all three appeared to invite causual interpretations. Compared to other types of persuasive impact, causal interpretations seem the most plausible for SDH; both The Commission and *Unnatural Causes* emphasized how poor health outcomes are a function, at least in part, of SDH. Moreover, the focus on relationships involving two or three variables belie challenges associated with describing complex interactions among factors that shape health outcomes and disparities. Visual representations of health determinants, while potentially helping synthesize complex data into relatively easy-to-understand formats, can missrepresent multivariate relationships by focusing on a few variables at a time [37]. Initial research in this area suggests that unlike narratives, images are not as able to effectively portray causal relationships between SDH and health (*i.e.*, why education status might be related to health). As a result, people may speculatively discuss why these relationships might exist and what other variables may be missing from the equation. Future research should therefore explore the persuasive effects of SDH-related narratives both with and without accompanying visual evidence. It is possible that narratives could illustrate broader trends offered by visual evidence presented in graphical and other forms. 

## 5. Conclusions

Our analysis of narratives and visual images disseminated by two national SDH awareness initiatives revealed a focus on certain types of SDH (including income/wealth and one’s home or and workplace environment) over other determinants; an emphasis on solutions for addressing these health issues that involve a “mixed” combination of self-driven motivation (such as changes in personal health behaviors) along with externally-driven factors such as government policy; and the use of images, especially graphs and charts, to suggest causal connections among SDH, health outcomes, and other variables (like the relationship between mother’s education and infant mortality, or the link between heart disease and education levels within communities).

Our results have implications for messages that communicate the importance of SDH and the need for policy interventions to address their impacts. Future work should explore how narrative and image characteristics used by The Commission and *Unnatural Causes* are perceived by both the public and policymakers, focusing on the potential challenges we have identified here.
